# Socioeconomic inequality in congenital heart diseases in Iran

**DOI:** 10.1186/s12939-021-01591-3

**Published:** 2021-12-04

**Authors:** Mostafa Amini-Rarani, Sajad Vahedi, Maryam Borjali, Mehdi Nosratabadi

**Affiliations:** 1 Health Management and Economics Research Center, Isfahan University of Medical Sciences, Isfahan, Iran; 2 Department of Health Care Management, School of Public Health, Ahvaz Jundishapour University of Medical Sciences, Ahvaz, Iran; 3 Department of Health and Social Welfare, School of Management and Medical Information Sciences, Isfahan University of Medical Sciences, Isfahan, Iran; 4 Social Determinants of Health Research Center, Isfahan University of Medical Sciences, Isfahan, Iran

**Keywords:** Concentration index, Congenital heart diseases, Decomposition, Health care inequality, Socioeconomic factors

## Abstract

**Introduction:**

Social-economic factors have an important role in shaping inequality in congenital heart diseases. The current study aimed to assess and decompose the socio-economic inequality in Congenital Heart Diseases (CHDs) in Iran.

**Methods:**

This is a cross-sectional research conducted at Shahid Rajaie Cardiovascular Medical and Research Center in Tehran, Iran, as one of the largest referral heart hospitals in Asia. Data were collected primarily from 600 mothers who attended in pediatric cardiology department in 2020. The polychoric principal component analysis (PCA) and Errygers corrected CI (ECI) were used to construct household socioeconomic status and to assess inequality in CHDs, respectively. A regression-based decomposition analysis was also applied to explain socioeconomic-related inequalities. To select the explanatory social, medical/biological, and lifestyle variables, the chi-square test was first used.

**Results:**

There was a significant pro-rich inequality in CHDs (ECI = -0.65, 95% CI, − 0.72 to − 0.58). The social, medical/biological, and lifestyle variables accounted for 51.47, 43.25, and 3.92% of inequality in CHDs, respectively. Among the social variables, family SES (about 50%) and mother’s occupation (21.05%) contributed the most to CHDs’ inequality. Besides, in the medical/biological group, receiving pregnancy care (22.06%) and using acid folic (15.70%) had the highest contribution.

**Conclusion:**

We concluded that Iran suffers from substantial socioeconomic inequality in CHDs that can be predominantly explained by social and medical/biological variables. It seems that distributional policies aim to reduce income inequality while increasing access of prenatal care and folic acid for disadvantaged mothers could address this inequality much more strongly in Iran.

## Background

Congenital Heart Diseases (CHDs) are structural or functional anomalies that occur during pregnancy. These disorders, also known as birth defects, congenital abnormalities, or congenital malformations, develop during pregnancy and can be discovered before, during, or after birth, as well as later in life. CHDs affect approximately 0.8 to 1.2% of live births [1, 2] with a global incidence rate of 17.9/1000 [3].

CHDs occur during fetal development and affect the infant from birth; they are among the most common birth defects, leading to childhood and infancy mortality [4]. The estimated prevalence of CHDs ranges from 4 to 50 per 1000 live births, and the difference in prevalence is primarily due to the age of diagnosis, the concept of CHDs, and the screening methods used [5].

Socioeconomic inequalities that disproportionately impact people of lower socioeconomic status (SES) over those of higher SES, have long been a cause of concern in cardiovascular health [6]. According to a cohort study from Eastern Europe in Russia, Poland and the Czech Republic, low socioeconomic status (SES) is a strong predictor of the high prevalence, morbidity, and mortality associated with cardiovascular diseases (CVD) and its main risk factors [7].

Health inequalities are more likely to be affected by socioeconomic factors than by medical and healthcare differences [8]. A minority of CHDs may be related to heritable or unintended genetic causes such as chromosomal abnormalities or pathogenic copy number variants (about 20%) [9]. Therefore, the vast majority of CHD incidents are believed to be caused by multifactorial causes, including various genetic, social and environmental factors [10, 11]. It has recently been shown that social determinants of health contribute to diseases and health outcomes and can lead to an increase in congenital anomalies [12, 13], including the formation of congenital heart disease [14]. In general, CHDs are related to serious difficulties such as cognitive impairment [15] and often affect parents and individuals disproportionately [16] in terms of mental health [17], quality of life and economic aspects [18].

It has been shown that different environmental and socioeconomic risk factors, as well as inequalities in social determinants of health, may play a significant role in the distribution and regional variations in the prevalence of CHDs. Few studies have looked at the importance of socioeconomic variables such as parental education/occupation and income as key outcome predictors in non-communicable diseases, which are considered to have a substantial effect on health and health inequalities [7], especially in the field of congenital heart disease [19]. In this regard, a study conducted in children born in Ontario, Canada (with access to public care) found that children born in families and neighborhoods with lower incomes and social status had significantly higher rates of congenital heart defects [20]. Thus, it has been shown that socioeconomic factors may be responsible for inequalities in the live-birth of children with CHDs [21]. Given that the prevalence of congenital heart diseases in Iran is between 9.7 and 17.5 per 1000 live births [22, 23], it remains uncertain if such socioeconomic factors among various groups are at proportionately higher risk of getting a child with congenital heart disease, especially in the Iranian context.

Furthermore, According to international organizations such as the World Bank [24] and UNICEF [25], inequalities in the distribution of external inputs such as education, income, wealth, and place of residence, which are beyond the control of children, contribute to inequality inopportunity for disadvantaged children [26, 27]. This form of socio-economic inequality is a major problem in developing countries, like Iran, where unfair socio-economic inequalities in early childhood lead to inequality in the health outcomes later in life [28, 29]. In other words, according to research, the prevalence of inequalities in access to health care to prevent physical and mental illnesses is dependent on the socio-economic situation of households [30, 31], and this type of unjust inequality would have negative consequences for the health and well-being of the children in these families. As a result, the central questions of this research are whether, in the area of congenital heart disease, the presence of socioeconomic inequality, which is a type of unfair inequality in parents’ health opportunities, would affect the prevalence of congenital heart disease in children, and what proportion of these inequalities in the birth of a child with congenital heart disease can be due to various socio-economic factors of the parents?

## Methods

### Setting

This is a cross-sectional study conducted at Shahid Rajaie Cardiovascular Medical and Research Center in Tehran, Iran, during March 2020 to September 2020. The hospital is one of the largest referral heart hospitals in Asia founded in 1976. It currently has over 600 active beds, and 1000 patients are admitted daily from various parts of Iran. This well-known, specialized and referral hospital welcomes a wide variety of clientele from different socioeconomic groups across Iran’s provinces One of the most important clinical services provided by this hospital is pediatric cardiac care. The pediatric cardiology department is a pioneer in educational, treatment, and research services, as well as the country’s most well-known clinic for heart diseases in children and adolescents. The department has provided 138 patient beds. This center is the largest and best-equipped hospital in the Middle East for treating the children with heart diseases. About 1400 congenital heart surgeries are performed in the pediatric cardiology department each year [32].

### Data collection

The data were collected primarily from the mothers who attended the pediatric cardiology department during April 2020 to June 2020. The mothers were chosen through convenience random sampling. They were asked to complete a research administrated check list including three types of variables: social, medical/biological, and lifestyle. The social and life style variables were selected based on the WHO framework of social determinants of health presented by Solar and Irwin [33]. The medical/biological variables were selected according to the previous studies, as well [10, 11, 34].

### Definition of variables

In this study, congenital heart diseases (CHDs) was chosen as a dichotomous outcome variable (mothers have had a child with CHDs or not).

Household socio-economic status, mother’s and father’s education levels, mother’s and father’s occupations, nationality, place of residence, number of children, and history of family marriage were all explanatory social variables. Explanatory medical/biological variables included mother’s age at delivery time, father’s age (as biological variables), parity number, receiving pregnancy care, mother’s history of abortion, chronic disease, and using folic acid (as medical variables). In addition, life style variables were as follows: doing physical activity, using alcohol, and smoking.

In the absence of direct data on income and expenditures, a common and widely used method for assessing the socioeconomic status of a household is to use an asset index (as a proxy of socio-economic status) from data on household ownership of durable assets and characteristics of the house [35]. The polychoric principal component analysis (PCA) was used to construct household socioeconomic status [36]. One of the assumptions underlying the classic PCA is that the input variables are normal. As our data included in household socio-economic status were discrete (including binary and ordinal variables), this assumption was clearly violated. As a result, polychoric PCA was used. The following variables were used in polychoric PCA model: mother’s education level, father’s education level, father’s occupation, house ownership, owning a personal computer, and having a kitchen, a bathroom, a vacuum cleaner, a washing machine, and a freezer. Accordingly, five socioeconomic quintiles including the poorest, poorer, middle, richer, and the richest were made and applied in the subsequent analyses. Mother’s age at delivery was also classified into four age categories [37] (≤17, 18–28, 29–39, and ≥ 40 years old).

### Inequality measurement and decomposition

Socioeconomic inequality in CHDs was measured using the Concentration Index (CI) [38]. The CI proposed by Wagstaff et al. (2003) has been widely applied to assess socio-economic inequality in health outcomes. Furthermore, the decomposition potential of CI has led to its widespread adoption as a reliable measure of health inequality rather than other measures of inequality [39].

The CI is defined using a Concentration Curve (CC) (1). The CC plots the cumulative percentage of the health outcome (Y axis) against the cumulative percentage of individuals, ranked by their socioeconomic status from the poorest to the richest (X axis). If everyone, irrespective of his/her socioeconomic status, has exactly the same value as the health outcome, the CC will form a 45-degree line called equality line. But if the health outcome takes lower (or higher) values than the individuals with lower socioeconomic status, the CC will lie below (or over) the equality line. The CI is measured as twice the covariance of a health outcome and fractional rank of socioeconomic status divided by the mean health outcome, as follows:

1 $$\mathrm{CI}=\frac{2}{\overline{\mathrm{Y}}}\mathrm{Cov}\left({\mathrm{Y}}_{\mathrm{i}},{\mathrm{R}}_{\mathrm{i}}\right)$$

Where *Y*_*i*_ is the health outcome (CHDs) of the *i* ^th^ child, $\overline{Y}$ denotes CHDs mean, and *R*_*i*_ indicates the fractional rank of the *i* ^th^ child in terms of the index of their household’s socioeconomic status. The negative and positive values of CI indicate that CHDs’ inequality is unevenly concentrated in the worse-off and better-off children, respectively. Given that CHDs was considered as a binary variable in the current study, Errygers corrected CI (ECI) was applied to assess CHDs’ inequality more precisely [40].

2 $$\mathrm{ECI}=\frac{4\overline{Y}}{Y_{max}-{Y}_{min}}\mathrm{CI}$$

In which *Y*_*max*_ and *Y*_*min*_ are the maximum and minimum of CHDs, respectively, and CI is obtained from eq. 1.

To decompose, the chi-square test was first used to select the explanatory variables to be included in the decomposition model. In other words, we conducted the chi-square test on CHDs and each social, medical/biological, and life style variables, and then the significant values were entered in the decomposition model. Then, to examine the contribution of each social, medical/biological, and life style variables to CHDs’ inequality, a regression-based decomposition analysis was used as well. The CHDs were first explained using a Generalized Linear Model (GLM) (eq. 3) and then decomposed using eq. 4 as follows:

3 $${\mathrm{y}}_{\mathrm{i}}=\upalpha +\sum_{\mathrm{k}}{\upbeta}_{\mathrm{k}}{\mathrm{x}}_{\mathrm{k}\mathrm{i}}+{\upvarepsilon}_{\mathrm{i}}$$

4 $${\mathrm{ECI}}_{\mathrm{y}}=4\left[\sum_{\mathrm{k}}{\upbeta}_{\mathrm{k}}{\overline{\mathrm{x}}}_{\mathrm{k}}.{\mathrm{ECI}}_{\mathrm{k}}+\mathrm{G}{\mathrm{C}}_{\upvarepsilon}\ \right]$$

Where ${\mathrm{x}}_{{\mathrm{k}}_{\mathrm{i}}}$ is the set of k determinants (in binary form) of CHDs β_k_ indicates the coefficient obtained through GLM, ε_i_ is an error term, and GC_ε_ is the generalized CI for ε_i_. Equation (4) comprised of explained (or deterministic) and unexplained (or probabilistic) components. The absolute contribution of an explanatory social, medical/biological, and life style variable could be taken by estimating the explained component. The analysis was conducted using STATA/SE (version 14; Stata Corporation, College Station, TX, USA).

## Results

In this study, 600 mothers completed the survey, of whom 200 (33.33%) had a child suffering from CHDs. As shown in Table 1, most of the mothers were in the middle SES group, had a middle school education level, were 29–39 years of age, were housewives, Iranian, resided in urban areas, got pregnancy care, and used acid folic. Fortunately, most of the mothers did not have family marriage, a history of abortion, alcohol use and smoking. The results showed that 43.83 and 43.50% of the studied mothers had two pregnancies and had two children, respectively. Regarding fathers, the data indicated that most of them had higher education levels, were aged 36–40, and worked in non-clerical jobs. CHDs were more prevalent among the families with the poorest SES, illiterate mothers, mothers aged 18–28 years at delivery who had chronic diseases, fathers with high school education levels, fathers with non-clerical jobs, and fathers of 31–35 years of age.

**Table 1:** Summary statistics of studied mothers (*n* = 600) and Congenital heart diseases distribution

Variable		N (%)	Congenital heart diseases distribution (***n*** = 200)	
			N (%)	***P***-value^a^
***Social variable***				
Socio-economic status	Poorest	120 (20)	98 (16.33)	< 0.001
	Poorer	120 (20)	68 (11.33)	
	Middle	138 (23)	20 (3.33)	
	Richer	109 (18.17)	13 (2.17)	
	Richest	113 (18.83)	1 (0.17)	
Mother’s education	Illiterate	82 (13.67)	64 (10.67)	< 0.001
	Primary school	111 (18.50)	49 (8.17)	
	Middle school	144 (24.00)	36 (6.00)	
	High school	143 (23.83)	38 (6.33)	
	University	120 (20.00)	13 (2.17)	
Father’s education	Illiterate	33 (5.50)	20 (3.33)	< 0.001
	Primary school	51 (8.50)	36 (6.00)	
	Middle school	99 (16.50)	40 (6.67)	
	High school	173 (28.83)	55 (9.17)	
	University	244 (40.67)	49 (8.17)	
Mother’s occupation	Housewife	368 (61.33)	163 (27.17)	< 0.001
	Clerical jobs	137 (22.83)	14 (2.33)	
	Non-clerical jobs	95 (15.83)	23 (3.83)	
Father’s occupation	Clerical jobs	226 (37.67)	37 (6.17)	< 0.001
	Non-clerical jobs	333 (55.50)	152 (25.33)	
	Unemployed	41 (6.83)	11 (1.83)	
Nationality	Iranian	576 (96.00)	187 (31.17)	0.027
	Non-Iranian	24 (4.00)	13 (2.17)	
Place of residence	Urban	494 (82.33)	127 (21.17)	< 0.001
	Rural	106 (17.67)	73 (12.17)	
Number of children	One child	202 (33.67)	78 (13.00)	0.043
	Two children	261 (43.50)	87 (14.50)	
	> = 3 children	137 (22.83)	35 (5.83)	
History of family marriage	Yes	144 (24.00)	83 (13.83)	< 0.001
	No	456 (76.00)	117 (19.50)	
**Medical/biological variables**				
Mother’s age at delivery time	≤17 (yr)	10 (1.67)	10 (1.67)	< 0.001
	18–28 (yr)	202 (33.67)	89 (14.83)	
	29–39 (yr)	309 (51.50)	82 (13.67)	
	> = 40 (yr)	79 (13.17)	19 (13.17)	
Father’s age	17–25 (yr)	46 (7.67)	33 (5.50)	< 0.001
	26–30 (yr)	105 (17.50)	37 (6.17)	
	31–35 (yr)	135 (22.50)	59 (9.83)	
	36–40 (yr)	171 (28.50)	33 (5.50)	
	> 41 (yr)	143 (23.83)	38 (6.33)	
Parity number	1	171 (28.50)	55 (9.17)	0.431
	2	263 (43.83)	83 (13.83)	
	> = 3	166 (27.67)	62 (10.33)	
Pregnancy care	Have	478 (79.67)	101 (16.83)	< 0.001
	Not have	122 (20.33)	99 (16.50)	
History of abortion	Yes	140 (23.33)	114 (19.00)	< 0.001
	No	460 (76.67)	140 (23.33)	
Chronic disease	Have	195 (32.50)	119 (33.33)	< 0.001
	Not have	405 (67.50)	81 (13.50)	
Using folic acid	Yes	482 (80.33)	101 (16.83)	< 0.001
	No	118 (19.67)	99 (16.50)	
**Life style variable**				
Physical activity	Enough	159 (67.37)	103 (43.64)	< 0.001
	Not enough	77 (32.63)	71 (30.08)	
Using alcohol	Yes	122 (20.33)	97 (16.17)	< 0.001
	No	478 (79.67)	103 (17.17)	
Smoking	Yes	111 (18.50)	92 (15.33)	< 0.001
	No	489 (81.50)	108 (18.00)	

^a^ Chi-square test

Note: Since the parity number was not statistically significant, this variable was excluded in decomposition model

The ECI of CHDs was −0.65 (95% CI, − 0.72 to − 0.58), implying that CHDs were more concentrated among the families with low SES levels, and it was not equally distributed among the people with different SES levels.

**Fig. 1:**
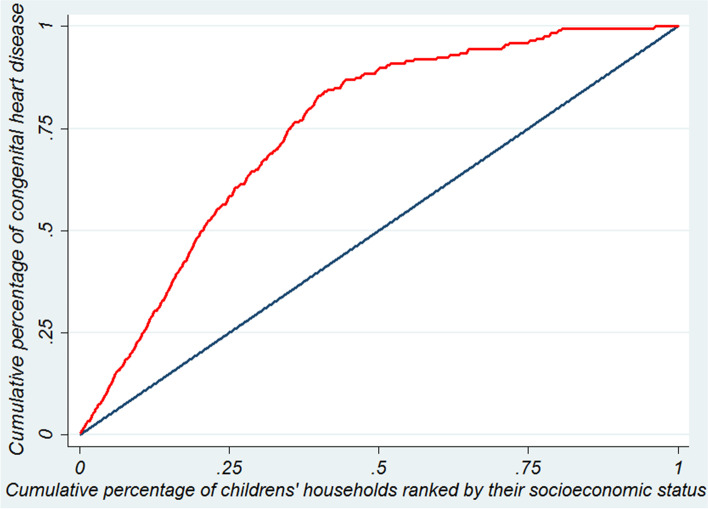
Concentration curve of congenital heart diseases

Figure 1 shows the concentration curve of CHDs. As it depicts, the concentration curve of CHDs lays above the equality line. This implies that CHDs were more concentrated among relatively lower SES families.

The decomposition results of SES inequality in CHDs are presented in Table 2. The ECIs of the explanatory variables revealed that mothers’ and fathers’ low educational attainment, being a housewife, father’s non-clerical job, having a history of family marriage, younger maternal age (< 28 years) and fathers’ age (< 25 years), having a history of abortion and chronic disease, using alcohol, and smoking were more concentrated among families with lower SES. In contrast, mothers’ and fathers’ clerical jobs, living in urban areas, mother’s age of 29–39 at delivery time, using prenatal care and acid folic as well as doing enough physical activity were all more concentrated among higher SES families. As Table 2 shows, the observed component including social, medical/biological, and life style variables accounted for 51.47, 43.25, and 3.92% of inequality in CHDs, respectively. The observed component indicated that the determinants included in the current model were able to explain 98.64% of the measured inequality in CHDs. The rest of the inequality (1.36%) was due to the residual (unexplained) component. The main contributors to inequality in CHDs regarding social variables were devoted to family SES (about 50%), mother’s occupation (21.05%), and mother’s education (3.62%). Besides, in the medical/biological group, receiving pregnancy care (22.06%), using acid folic (15.70%), and a history of abortion (5.38%) were the most contributors to inequality in CHDs. In addition, regarding the life style variables, using alcohol and smoking accounted for 2.67 and 1.87% of inequality in CHDs, respectively. About the contributing variables, if the value of the contributor X was *x* and positive (or negative), the inequality in CHDs would decrease (or increase) by *x*% if the contributor was to become equally distributed among different socioeconomic groups. The largest contribution to inequality in CHDs was attributed to family SES (about 50%), i.e. if income and wealth were equally distributed in the society, the inequality in CHDs would decrease by 50%. Also, if employment opportunities and access to pregnancy care were equally distributed among different mothers, the inequality in CHDs would decrease by 21 and 22%, respectively.

**Table 2:** Decomposition of concentration index for congenital heart disease in Iran

		β	Mean	ECI	Absolute Contribution	Contribution (%)
***Social variables***						
Socio-economic status	Poorest	0.59	0.20	−0.57	− 0.27	41.39
	Poorer	0.55	0.20	−0.28	− 0.12	18.95
	Middle	0.27	0.23	0.05	0.01	−1.91
	Richer	0.24	0.18	0.33	0.06	−8.77
	Richest	Ref	–	–	–	–
	Sum					49.66
Mother’s education	Illiterate	0.14	0.14	−0.25	−0.02	3.02
	Primary school	0.08	0.19	−0.21	− 0.01	1.96
	Middle school	0.01	0.24	−0.06	0.00	0.09
	High school	0.07	0.24	0.14	0.01	−1.45
	University	Ref	–	–	–	–
	Sum					3.62
Father’s education	Illiterate	−0.19	0.06	0.04	0.00	0.28
	Primary school	−0.21	0.09	−0.19	0.01	−2.21
	Middle school	−0.25	0.17	−0.28	0.05	−7.32
	High school	−0.10	0.29	−0.08	0.01	−1.43
	University	Ref	–	–	–	–
	Sum					−10.68
Mother’s occupation	Housewife	0.11	0.61	−0.40	− 0.11	16.52
	Clerical jobs	−0.08	0.23	0.40	−0.03	4.53
	Non-clerical jobs	Ref	–	–	–	–
	Sum					21.05
Father’s occupation	Clerical jobs	0.20	0.38	0.63	0.19	−29.46
	Non-clerical jobs	0.19	0.56	−0.38	− 0.16	24.88
	Unemployed	Ref	–	–	–	–
	Sum					−4.58
Nationality	Iranian	0.13	0.96	0.05	0.02	−3.84
	Non-Iranian	Ref	–	–	–	–
Place of residence	Urban	0.00	0.82	0.29	0.00	0.00
	Rural	Ref	–	–	–	–
Number of children	One child	−0.16	0.34	− 0.05	0.01	−1.67
	Two children	−0.09	0.44	0.06	−0.01	1.46
	> = 3 children	Ref	–	–	–	–
						−0.21
History of family marriage	Yes	−0.10	0.24	−0.24	0.02	−3.54
	No	Ref	–	–	–	–
***Total social variables***						***51.47***
***Medical/biological variables***						
Mother’s age at delivery time	≤17 (yr)	−0.01	0.02	−0.05	0.00	−0.01
	18–28 (yr)	0.04	0.34	−0.09	0.00	0.75
	29–39 (yr)	0.10	0.52	0.13	0.03	−4.16
	> = 40 (yr)	–	–	–	–	–
	Sum					−3.41
Father’s age	17–25 (yr)	0.17	0.08	−0.12	−0.01	1.00
	26–30 (yr)	0.15	0.18	0.07	0.01	−1.16
	31–35 (yr)	0.13	0.23	−0.09	− 0.01	1.66
	36–40 (yr)	−0.02	0.29	0.09	0.00	0.32
	> 41 (yr)	Ref	–	–	–	–
	Sum					1.82
Pregnancy care	Have	−0.16	0.80	0.28	−0.14	22.06
	Not have	Ref	–	–	–	–
History of abortion	Yes	0.10	0.23	−0.38	− 0.03	5.38
	No	Ref	–	–	–	–
Chronic disease	Have	0.03	0.33	−0.28	− 0.01	1.71
	Not have	Ref	–	–	–	–
Using folic acid	Yes	−0.11	0.80	0.29	−0.10	15.70
	No	Ref	–	–	–	–
***Total Medical/biological variable***						***43.25***
***Life style variables***						
Physical activity	Enough	0.01	0.67	0.15	0.00	−0.62
	Not enough	Ref	–	–	–	–
Using alcohol	Yes	0.07	0.20	−0.31	− 0.02	2.67
	No	Ref	–	–	–	–
Smoking	Yes	0.05	0.19	- 0.32	−0.01	1.87
	No	Ref	–	–	–	–
**Total life style**						***3.92***
**Total observed**					**−0.64**	**98.64**
**Residual**					−0.01	1.36
**Total**					**−0.65**	**100.00**

## Discussion

Previous studies have shown that disadvantaged groups have a higher rate of CHDs' morbidity and mortality [19]. The current research was carried out in order to provide some evidence regarding social inequalities in CHDs and the factors that played crucial roles in shaping such unwanted inequality. To the knowledge of the researchers, the present study is the first research that measured such inequality through concentration index and explained it via regression-based decomposition analysis in the globe. We found substantial socioeconomic inequality in CHDs which was in favor of the better-offs. Although there were no related studies, other studies showed that other negative outcomes such as preterm birth and still birth [41] were concentrated among the disadvantaged groups. Unfortunately, it seems that socioeconomic inequality in mortality due to CHDs increased across the world over time [42]. As children have no choices about their health status, our findings implicitly indicated huge inequality of opportunities for CHDs’ patients within the health system of Iran and probably other low- and middle-income countries. Furthermore, it seems that survived children with congenital anomalies such as heart disease will have less opportunities to improve their health resources [43].

The explanation of socioeconomic inequalities has an intrinsic value and could assist policy makers to address these inequalities much effectively [44]. The decomposition analysis revealed that socioeconomic factors solely explained about 51% of the observed inequality in CHDs. A study conducted through mediation analysis in the US also found that socioeconomic factors such as education and health insurance accounted for a significant portion of racial and ethnic disparities in CHDs [45]. Surprisingly, income status contributed somewhat further to socioeconomic inequality in our analysis and could be considered as a key contributor. Previous research [21, 46, 47], found that lower socioeconomic status was substantially correlated to the risk of CHDs, which is consistent with our findings, but those studies failed to trace the impact of this predictor on socioeconomic inequality in CHDs. However, other studies showed that economic status was the main contributor to socioeconomic inequality in infants and children mortality [48, 49]. Furthermore there is some evidence that shows income inequality has negative effects on cardiovascular disease in the US [50] and globally on heart failure [51]. Parents with higher incomes are able to make more investments in their health capital through purchasing medical care services, nutritious food, and other health-related products, as well as providing safer environments. Hence, children of these parents are likely to be healthier comparing to those of lower-income parents [52]. Alongside socio-economic status, the effect of parental characteristics on CHDs’ inequality was also studied. We observed that although mother’s occupation positively contributed to CHDs’ inequality, father’s occupation was negatively contributed to this outcome. Housewives accounted for the greatest share of CHDs’ inequality within mother’s occupation category (16.52%).It seems that housewife mothers do not have enough affordability to experience safe pregnancy and this could increase CHDs among their children [53].

Although, clerical and non-clerical employed father approximately neutralized each other effects on CHDs’ inequality, but given to the positive contribution of father’s non-clerical occupation in CHDs’ inequality, it seems that non-clerical occupation groups suffer from insufficient income, job insecurity, lack of medical insurance and access to healthcare [54]. Besides, Occupation status could also associate with CHDs through occupational exposure to chemicals [55] and this could be increased among parents with non-clerical jobs which seemed to be more prevalent among disadvantaged households. So, it could be said that more attention should be paid to the households that their fathers have non-clerical jobs.

In the current study, the father’s age had only a small contribution to CHDs’ inequality but, it might work as a social factor from the point of view of occupation, income (e.g. mean to pay) and/or knowledge and experiences.

The decomposition analysis in this study was not limited to socioeconomic factors; the effects of other variables such as medical/biological and lifestyle variables were also investigated. Second to socioeconomic variables, medical/biological variables played a crucial role in shaping socioeconomic inequality in CHDs through their positive contribution to inequality. Among the variables of this category, using pregnancy care and folic acid collectively explained 38% of the observed inequality. There are well-established evidence about the protecting effect of pregnancy care such as prenatal screening and the use of folic acid against CHDs [56, 57]. It is believed that the misuse of pregnancy services and supplements by disadvantaged mothers could be an important justification of the association between lower socioeconomic status and the risk of CHDs [58]. These factors in our analysis had negative concentration index indicating that disadvantaged households had undesirable access to pregnancy care and folic acid. Iran has been subject to international sanctions over the years, which are believed to have negatively affected food security and access to the required healthcare services [59]. Hence it seems that Iranian healthcare authorities could be able to address remarkable of CHDs inequality through facilitating the access of disadvantaged mothers to the pregnancy care and folic acid this could be done through providing subsidized prenatal care and including accessing to the folic acid in national food fortification strategy. Life style factors also had positive contribution on the CHDs’ inequality in our research that was in line with former studies [54]. We observed that the dangerous life style behaviors such as using alcohol and smoking were concentrated among the poor. Hence, health promotion interventions among disadvantaged groups could decrease the CHDs’ inequality to some extent. The children with CHDs have higher hospitalization rates with expensive healthcare services [56]. According to our findings, the disadvantaged households that have children with CHDs were in a higher risk of experiencing financial hardship [60]. As a result, it is strongly recommended that healthcare authorities place these households at the core of their policy-making.

### Strengths and limitations

Although our study was the first research that simultaneously measured and explained socioeconomic inequality in the area of CHDs through gathering primary data, several limitations need to be acknowledged. First, the results of the present research are based on cross-sectional data and hence, could not bring strong causality between the studied variables and CHDs’ inequality. Second, despite the fact that this analysis examined the CHDs’ inequality using a broad variety of variables, the majority of those variables were social in nature; however, there are some others that we did not address in this report, including parental occupational exposure to chemicals or clinical variables. Third, there are concerns about sample bias with this study, as this study conducted at Shahid Rajaie Cardiovascular Medical and Research Center. It is possible that a number of families and mothers whose children might have CHDs but for various reasons did not have access to diagnosis/care. Accordingly, neglected patients might suffer and/or lose their lives and could not be included in this study.

## Conclusion

This research tried to firstly investigate determinants of socioeconomic inequality in CHDs. We showed that Iran suffers from remarkable pro-rich inequality in CHDs. Considering that social variables including socioeconomic status and mother’s occupation are the main contributor to the CHDs’ inequality, policy makers must pay special attention to these social conditions in which children are born and grow. They can try to provide equal opportunities to decrease the occurrence of CHDs, its inequality, and other related unjustified outcomes. The first policy entry point to tackle CHDs’ inequality is to promote an equitable distribution of income across various SES groups. Second, it is recommended to develop a comprehensive social program and provide special health services for housewife mothers. Third, in terms of medical contributors to the CHDs’ inequality, it seems that providing subsidized prenatal care and national food fortification strategy can address the CHDs’ inequality among disadvantaged households and the burden in the health system in general. Moreover, it seems that addressing the social and medical factors contributing to the CHDs’ inequality, simultaneously, can further improve the equality in the CHDs’ status quo.

## Data Availability

Data are available on reasonable request. The data that support the findings of this study are available from the corresponding author.
